# C-Reactive Protein as a Negative Predictive Marker for Anastomotic Leakage After Minimally Invasive Esophageal Surgery

**DOI:** 10.1007/s00268-023-07013-5

**Published:** 2023-04-27

**Authors:** Eliza R. C. Hagens, Minke L. Feenstra, Wing C. Lam, W. J. Eshuis, W. Lameris, Mark I. van Berge Henegouwen, Suzanne S. Gisbertz

**Affiliations:** Department of Surgery, Cancer Center Amsterdam, Amsterdam UMC, University of Amsterdam, Meibergdreef 9, 1105 AZ Amsterdam, The Netherlands

## Abstract

**Background:**

Serum C-reactive protein (CRP) is commonly used by surgeons to raise suspicion of anastomotic leakage and other infectious complications, but most studies on optimal cut-off values are retrospective with a small sample of patients. The aim of this study was to determine the accuracy and optimal cut-off value of CRP for anastomotic leakage in patients following esophagectomy for cancer.

**Materials and methods:**

Consecutive minimally invasive esophagectomy for esophageal cancer patients was included in this prospective study. Anastomotic leakage was confirmed if a defect or leakage of oral contrast was seen on a CT scan, by endoscopy or if saliva was draining from the neck incision. Diagnostic accuracy of CRP was assessed by receiver operator curve (ROC) analysis. Youden’s index was adopted to determine the cut-off value.

**Results:**

A total of 200 patients were included between 2016 and 2018. Postoperative day 5 showed the highest area under the ROC (0.825) and optimal cut-off value of 120 mg/L. This resulted in a sensitivity of 75%, specificity of 82%, negative predicting value of 97%, and positive predicting value of 32%.

**Conclusions:**

CRP on postoperative day 5 can be used as a negative predictor for and can be used as a marker to raise suspicion of anastomotic leakage following esophagectomy for esophageal cancer. When CRP exceeds 120 mg/L on postoperative day 5, additional investigations should be considered.

## Introduction

Anastomotic leakage following esophagectomy for cancer is a severe complication, generally leading to additional postoperative morbidity or even death [1–4]. C-reactive protein (CRP) is a serum acute-phase reactant produced by the liver in response to pro-inflammatory cytokines that play a role in activating the complement system [5]. CRP can be tested easily at low cost and is, therefore, often used to detect infectious complications after surgery [6]. In colorectal surgery, it has been validated as a useful negative predictor test for septic complications, and cut-off values have been determined as guidance for additional diagnostic tests [7]. CRP is used by most surgeons to detect postoperative complications after esophagectomy, but the literature on optimal cut-off values are retrospective with a small sample of patients [7–10]. Identifying an optimal cut-off value for CRP for anastomotic leakage may contribute to earlier detection of a complicated postoperative course and can be used in ERAS protocols to guide the indication for additional investigations such as a CT scan or endoscopy.

The primary aim of this study was to determine the accuracy and optimal cut-off values of CRP with regard to anastomotic leakage in esophageal cancer patients following minimally invasive esophagectomy. The secondary aims were to determine if there is an association between the level of CRP and the severity of anastomotic leakage and the correlation between the level of CRP and incidence of other complications.

## Materials and methods

This prospective observational study was conducted in the Amsterdam UMC, location AMC, the Netherlands. According to national guidelines, this study was exempted from official institutional review board (IRB) approval. The IRB assessed the study design and protocol, and a waiver was obtained. Informed consent was obtained from all patients. The Standards for Reporting Diagnostic accuracy studies (STARD) guidelines were used to ensure the correct reporting of this diagnostic study [11].

### Study population

Consecutive patients with a histologically proven, resectable (cT0-4aN0-3M0) esophageal or gastroesophageal junction carcinoma in whom a minimally invasive esophagectomy with gastric conduit reconstruction was performed between April 2016 and October 2018 were included. Patients were excluded if a salvage esophagectomy was performed.

### Surgery and postoperative CRP measurements

Esophagectomy was performed by means of a minimally invasive, formation of a gastric conduit, and construction of an intrathoracic or cervical anastomosis. The indication for a cervical anastomosis depended on the location of the primary tumor (≧ mid-esophageal), the clinical presence of paratracheal lymph node metastases, and the proximal extension of the radiation field (with the aim of creating the anastomosis outside the radiation field). A two-field lymphadenectomy was performed (Stations 2 on indication, 4, 5, 7, 8, 9, and 15–19 according to the AJCC 8th edition and lymph nodes in the hepatoduodenal ligament). Levels of CRP were measured on postoperative days 3, 5, and 7, according to local protocol. There may be reasons for deviating from this protocol, such as for clinical deterioration warranting determining of CRP on an earlier day or admittance to the ICU, where CRP was not routinely measured.

### Postoperative complications

The diagnosis of anastomotic leakage was made when a defect or leakage of oral contrast was seen on a CT scan or by endoscopy or if saliva was draining from the neck incision. Final confirmation of diagnosis was made with consensus by all upper GI surgeons (SSG, MvBH, and WJE). The day of diagnosis is defined as the day anastomotic leakage was confirmed on a CT scan, by endoscopy of saliva was draining from the neck incision. Definitions and grading of anastomotic leakage and other complications were done according to the Esophageal Complications Consensus Group (ECCG) classification [12]. A type 1 leak was defined as a local defect requiring no change in therapy or treated medically or with dietary modification, a type 2 leak includes a localized defect requiring interventional but not surgical therapy, for example, interventional radiology drain, stent or bedside opening, and packing of incision, and a type 3 leak was defined as a localized defect requiring surgical therapy [12].

### Outcome parameters

Primary outcomes were the accuracy of CRP as a marker for anastomotic leakage and the optimal cut-off value of CRP to detect anastomotic leakage. Secondary study outcomes included the association between the severity (grade) of anastomotic leakage and the level of CRP, and the correlation between CRP and other complications.

### Sample size calculation

The aim was to include a minimal number of events (patients who develop anastomotic leakage after surgery) of 20 in this study, since this is a generally accepted number of events for the calculation of the diagnostic accuracy of a test [13]. The incidence of anastomotic leakage in the literature varies from 3 to 25% [14, 15]. An incidence of 10% was used because the incidence of anastomotic leakage is around 10% in the study center [16]. Based on these numbers, it was determined that at least 200 patients needed to be included in this study.

### Statistical analysis

Continuous variables with normal distributions are presented as means with standard deviations and were compared using independent *t*-test. Medians and interquartile ranges (IQR) were used as central tendency for continuous variables with non-normal distributions, these data were compared using the Mann–Whitney *U*-test. Categorical data were expressed with percentage frequencies and were compared using a Chi-square or Fisher’s exact test where appropriate. Difference in CRP levels between patients with and without complications was analyzed using the independent *t*-test. The diagnostic accuracy of CRP was assessed by receiver operator curve (ROC) analysis for CRP on postoperative days 3, 5, and 7. The area under the ROC (AUC) is a direct measure of the diagnostic accuracy of a test. An AUC value > 0.50 indicates the ability of a test to significantly discriminate between positive and negative cases with regard to the classification variable (e.g., presence or absence of disease). Youden’s index was adopted to determine the cut-off value in ROC analysis with highest sensitivity and specificity. Sensitivity, specificity, positive predictive value, and negative predictive value were calculated for these CRP cut-off values on postoperative days 3, 5, and 7. Kruskal–Wallis test was used to compare CRP levels in patients with different types of severity of anastomotic leakage. A *P*-value < 0.05 (two-sided tests) was considered significant. Missing data were handled with complete case analysis. Statistical analysis was performed with SPSS, version 24.0 for Windows (SPSS Inc., Chicago, IL).

## Results

There were 200 patients included in this study. In 13 patients (6.5%), an open esophagectomy was performed, these patients were excluded from analyses. Baseline characteristics are shown in Table 1. With 20 patients developing anastomotic leakage, the incidence was 11%. More patients in the anastomotic leakage group had diabetes. Twenty-six percent of the patients (6 out of 23) with a cervical anastomosis developed anastomotic leakage, significantly higher than the 8.5% of the patients (14 out of 164) with anastomotic leakage with an intrathoracic anastomosis (*p* = 0.011). Patients with anastomotic leakage were more often readmitted within 30 days (*p* < 0.001) and had a higher rate of in-hospital mortality (0 patients without anastomotic leakage died in-hospital and 2 out of 20 patients with anastomotic leakage died in-hospital, *p* = 0.011). The postoperative day of diagnosis of anastomotic leakage ranged from 1 to 15 days, with a median of 8 days.

**Table 1:** Baseline characteristics of all patients, and patients with and without anastomotic leakage

	All patients	No anastomotic leakage	Anastomotic leakage	
	*n* = 187	*n* = 167	*n* = 20	*p* value
Male gender	154 (82.4)	138 (82.6)	16 (80.0)	0.770
Age (years) median (IQR)	66 (59–71)	65 (59–71)	68 (62–70)	0.110
BMI (kg/m^2^) median (IQR)	25 (23–28)	25 (23–27)	26 (25–31)	0.055
Comorbidity				
Cardiovascular	97 (51.9)	84 (50.3)	13 (65.0)	0.244
COPD	8 (4.3)	7 (4.2)	1 (5.0)	0.866
Diabetes Mellitus type 2	26 (13.9)	20 (12.0)	6 (30.0)	**0.028**
ASA-classification				
I	53 (28.3)	48 (28.7)	5 (25.0)	0.598
II	95 (50.8)	86 (51.5)	9 (45.0)	
III	39 (20.9)	33 (19.8)	6 (30.0)	
Histology				
Adenocarcinoma	140 (74.9)	127 (76.0)	13 (65.0)	0.077
Squamous cell carcinoma	38 (20.3)	34 (20.4)	4 (20.0)	
Other	9 (4.8)	6 (3.6)	3 (15.0)	
cT-stage				
cT1	12 (6.4)	11 (6.6)	1 (5.0)	
cT2	42 (22.5)	38 (22.8)	4 (20.0)	1.000
cT3	128 (68.4)	113 (67.7)	15 (75.0)	
cT4	1 (0.5)	1 (0.6)	0	
cTx	3 (1.6)	3 (1.8)	0	
cTis	1 (0.5)	1 (0.6)	0	
cN-stage				
cN0	64 (34.2)	55 (32.9)	9 (45.0)	
cN1	84 (44.9)	77 (46.1)	7 (35.0)	0.756
cN2	34 (18.2)	30 (18.0)	4 (20.0)	
cN3	2 (1.1)	2 (1.2)	0	
cNx	3 (1.6)	3 (1.8)	0	
Neoadjuvant treatment				
None	19 (10.2)	17 (10.2)	2 (10.0)	0.851
Chemotherapy	13 (7.0)	11 (6.6)	2 (10.0)	
Chemoradiation	155 (82.9)	139 (83.2)	16 (80.0)	
Anastomosis				
Cervical (all manual)	23 (12.3)	17 (10.2)	6 (30.0)	**0.011**
Intrathoracic (all stapled)	164 (87.7)	150 (89.8)	14 (70.0)	
Hospital stay (days), median (IQR)	10 (8–15)	9 (8–13)	33 (18–41)	** <0.001**
Readmission within 30 days	28 (15.0)	19 (11.4)	9 (45.0)	** <0.001**
Mortality within 30 days	2 (1.1)	1 (0.6)	1 (5.0)	0.203
Hospital mortality	2 (1.1)	0	2 (10.0)	**0.011**

Bold values indicate statistical significance

Data presented as* n*(%), unless indicated otherwise

* IQR* interquartile range,* BMI* body mass index,* COPD* chronic obstructive pulmonary disease,* ASA* American Society of Anaesthesiologists, and* MIE* minimally invasive, TNM staging according to AJCC 8th edition

### Optimal cut-off value and accuracy of CRP to detect anastomotic leakage

**Fig. 1:**
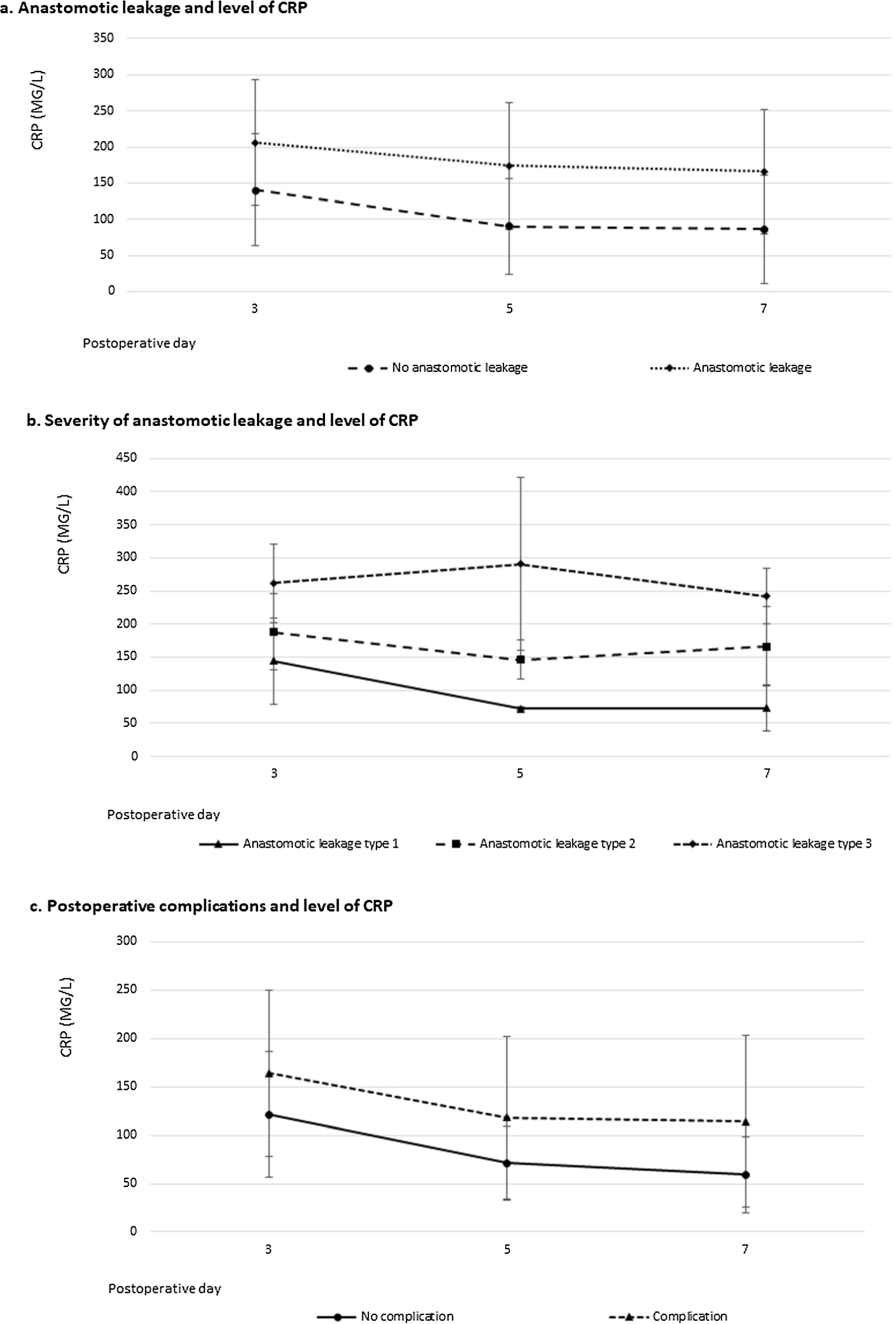
Median levels of CRP on postoperative days 3, 5, and 7 in patients with and without complications. **a** Anastomotic leakage and level of CRP (*data displayed as mean with standard deviation*). b Severity of anastomotic leakage and level of CRP (*data displayed as median with interquartile range).* **c** Postoperative complications and level of CRP (*data displayed as mean with standard deviation)*

CRP was measured in 174 patients on day 3, in 158 patients on day 5, and in 128 patients on day 7. The mean level of CRP for patients with and without anastomotic leakage was 206 and 141 mg/L, respectively, on day 3 (*p* < 0.001), 174 and 91 mg/L on day 5 (*p* < 0.001), and 166 and 86 mg/L on day 7 (*p* < 0.001). Figure 1a shows the levels of CRP on postoperative days 3, 5, and 7 for patients with and without anastomotic leakage.

Table 2 shows different cut-off values for CRP on postoperative days 3, 5, and 7, and corresponding AUC, sensitivity, specificity, positive predicting value, and negative predicting value. The level of CRP on postoperative day 5 has the highest accuracy to detect anastomotic leakage, with a sensitivity of 75%, specificity of 82%, negative predicting value of 97%, and positive predicting value of 32%. The optimal cut-off value for day 5 was 120 mg/L.

**Table 2:** CRP cut-off values and diagnostic values on POD 3, 5, and 7 for all complications and anastomotic leakage

	Cut-off value CRP (mg/L)^a^	AUCROC (95% CI)	*P*-value^1^	Sensitivity % (95% CI)	Specificity % (95% CI)	PPV% (95% CI)	NPV% (95% CI)
Diagnostic values for prediction of any postoperative complication in all patients							
CRP POD-3^b^ CRP POD-5^c^ CRP POD-7^d^	100 120 72	0.653 (0.570–0.736) 0.678 (0.595–0.761) 0.696 (0.605–0.786)	**0.001** ** < 0.001** ** < 0.001**	76 (68–84) 34 (24–44) 61 (51–72)	51 (39–63) 92 (86–99) 73 (58–84)	71 (63–79) 86 (75–98) 80 (70–90)	57 (44–69) 49 (40–58) 50 (38–62)
Diagnostic values for prediction of anastomotic leakage in all patients							
CRP POD-3^b^ CRP POD-5^c^ CRP POD-7^d^	141 120 137	0.733 (0.628–0.832) 0.825 (0.735–0.916) 0.789 (0.669–0.903)	**0.001** ** < 0.001** ** < 0.001**	79 (61–97) 75 (54–96) 71 (48–95)	61 (53–68) 82 (76–89) 83 (76–90)	20 (11–29) 32 (17–48) 34 (17–52)	96 (92–100) 97 (94–100) 96 (92–100)

Bold values indicate statistical significance

*CRP* C-reactive protein, *POD* postoperative day, *NVW* negative predicting value, *PVW* positive predicting value, *CI* confidence interval

^a^Based on highest Youden index, ^p1^comparison between AUCROC and reference line, ^b^based on 174 patients, ^c^based on 158 patients, ^d^based on 128 patients, ^e^based on 154 patients, ^f^based on 142 patients, and ^g^based on 107 patients because in others CRP measurement failed on this day

### Severity of anastomotic leakage and level of CRP

Although the median level of CRP in patients with a more severe grade of anastomotic leakage was higher, the severity of anastomotic leakage did not significantly correlate with the level of CRP (Table 3). There was also no significant difference between the level of CRP and grade of anastomotic leakage when comparing type 1 leakages with types 2 and 3 leakages together (the median levels of CRP for patients with a type 1 or type 2/3 leak were 144 and 204 mg/L, *p* = 0.159 on day 3; 72 and 160 mg/L, *p* = 0.101 on day 5; and 73 and 200 mg/L, *p* = 0.122 on day 7). Figure 1b shows the level of CRP for the different types of anastomotic leakage.

**Table 3:** Median CRP level for different types of anastomotic leakage

	No anastomotic leakage	Anastomotic leakage type 1	Anastomotic leakage type 2	Anastomotic leakage type 3	*P* value^1^
CRP POD-3	120 (15–399)	144 (79–174)	188 (101–398)	262 (188–300)	0.122
CRP POD-5	73 (15–348)	72 (69–163)	146 (86–321)	291 (160–312)	0.120
CRP POD-7	63 (11–429)	73 (38–165)	166 (65–347)	242 (200–285)	0.132

Data displayed as median (range) in mg/L. Type of anastomotic leakage according to ECCG. ^p1^Patients without anastomotic leakage were excluded from this analysis.* POD* postoperative day

### Level of CRP and other complications

One hundred and nineteen patients (64%) had at least one postoperative complication, and 36 of these patients (30%) had a complication of Clavien–Dindo grade IIIB or higher. Fifty patients (27%) had a pulmonary complication, 56 (30%) had a cardiac complication, 23 patients (12%) had chyle leakage, 6 (3%) of the patients had a urologic complication, 5 patients (3%) had vocal cord paresis, and 31 patients (17%) had another complication.

The mean level of CRP for patients with and without any complication was 164 and 121 mg/L, respectively, on day 3 (*p* < 0.001), 118 and 71 mg/L on day 5 (*p* < 0.001), and 114 and 59 mg/L on day 7 (*p* < 0.001). Figure 1c shows the mean level of CRP for patients with any complication or no complication. Table 2 shows the optimal cut-off values for CRP on postoperative days 3, 5, and 7, and corresponding AUC, sensitivity, specificity, positive predicting value, and negative predicting value for predicting any postoperative complication. The level of CRP on postoperative day 7 had the highest AUC (0.696), with an optimal cut-off value of 72 mg/L.

## Discussion

The primary aim of this prospective observational study was to determine the accuracy and optimal cut-off values of CRP to predict anastomotic leakage. We found that the optimal postoperative day to predict anastomotic leakage was day 5, with a cut-off value of 120 mg/L. This resulted in an AUC of 0.825, sensitivity of 75%, specificity of 82%, and a positive predictive value and negative predictive value of 32 and 97%, respectively. This is one of the few studies reporting about the correlation between CRP and anastomotic leakage after minimally invasive esophageal surgery and determining an optimal cut-off value for CRP as guidance for further diagnostic tests.

The level of CRP on postoperative day 5 had the highest accuracy to detect anastomotic leakage with an optimal cut-off value of 120 mg/L. Most other studies also found postoperative day 5 the most accurate day to measure the level of CRP. Nonetheless, the cut-off value of 120 mg/L is lower than in other studies where the cut-off levels range from 154 to 189 mg/L [17–20]. Authors of these studies used similar statistical techniques to determine the optimal cut-off value. Most of these studies, however, were retrospective in design and contained a small sample of patients, with a wider variety of surgical approaches. Only one study, by Asti et al., found a lower optimal cut-off value than the present study, this was a CRP level of 83 mg/L [21]. A reason for the low cut-off value of CRP in both cohorts could be that all patients were operated by minimally invasive approach. More invasive esophageal surgery, such as an open esophagectomy, is correlated with a higher level of CRP posteroperatively [22].

The diagnostic accuracy of CRP on postoperative day 5 was high, with an AUC of 0.825. With a cut-off value of 120 mg/L, specificity and negative predicting value were 82 and 97%, respectively, while sensitivity and positive predicting value were lower (75 and 32%). Other studies also found higher specificity and negative predicting values compared to sensitivity and positive predicting values [23]. This indicates that CRP is a feasible marker to guide the use of additional investigations such as CT scanning to detect anastomotic leakage. In case of a low CRP, anastomotic leakage becomes very unlikely, and clinical observation can be continued.

Although the CRP level was directly proportional with the severity of anastomotic leakage, this was not statistically significant. Due to the small number of patients in the different severity groups, there might have been insufficient power to reach significance. Another possibility could be that not the level of CRP itself, but the rapidity of rise in CRP could be an indicator for the severity of the leakage. An indicator to predict the severity of anastomotic leakage can be of clinical importance, as it could contribute to decision making in the management of leakage. Unfortunately, the number of patients was too small to look at the influence of difference of CRP level between days 3 and 5 on the severity on the leakage. Moreover, there is no available evidence to compare our results with.

CRP is a marker of inflammation, and elevated levels of CRP can be caused by other inflammatory conditions, for example, pneumonia. CRP is not specific for anastomotic leakage, and other clinical symptoms should, therefore, be considered when evaluating CRP. This makes interpretation of accuracy parameters difficult to interpret. However, CRP can be used as a guide for the conditional use of postoperative CT scanning or endoscopy. In the present study, 40 patients had a CRP of 120 mg/L or higher on day 5, but only 12 of these patients (30%) had anastomotic leakage. This suggests that 69% of the patients would have received “unnecessary” additional diagnostics. However, the majority of these patients had another complication that would have been likely to find on a CT scan. CRP is an unspecific marker, feasible to detect postoperative complications, including anastomotic leakage.

Four out of 16 patients with anastomotic leakage (and measurement of CRP available on day 5) did not have a CRP higher than 120 mg/L on day 5. It should be noted that in all of these patients, anastomotic leakage was detected on day 7 or later. It is possible that anastomotic leakage in these patients occurred later on than usual, and therefore, they did not have a CRP higher than 120 mg/L on day 5. Despite the high NPV of 96% in patients with a CRP < 120, additional CT scan should be performed in patients with (late) clinically suspected anastomotic leakage.

A limitation of the present study was that only CRP levels of days 3, 5, and 7 were routinely measured. Levels on more postoperative days could have resulted in a cut-off value with higher diagnostic accuracy. Also, not in every patient CRP levels were available for days 3, 5, and 7 which might have caused bias. Moreover, determining only on days 3–5–7 may be insufficient for early detection of anastomotic leakage, as this may occur on the in-between days. Moreover, it would have been valuable to stratify our results for cervical and intrathoracic anastomosis, since cut-off levels might be different. Though there were only 14 patients in the group of patients with an intrathoracic anastomosis and six patients in the group of patients with a cervical anastomosis. Unfortunately, these numbers of events are too low to perform a reliable analysis on these subgroups.

CRP can be a useful tool, but other symptoms such as fever can also indicate that there is an anastomotic leakage. Future studies should attempt to identify predictors for different types of anastomotic leakage and possibly stratify results for a cervical and intrathoracic anastomosis. Predicting anastomotic leakage might improve when other markers such as white blood cell count, temperature, or amylase level in drain fluid are also considered. A combination of different markers could contribute to a useful algorithm in the diagnosis and management of anastomotic leakage.

## Conclusion

CRP on postoperative day 5 can be used as a marker to raise suspicion of anastomotic leakage in patients following an esophagectomy for esophageal cancer, but can especially be used as a negative predictor. With a cut-off value of 120 mg/L, a negative predicting value of 97% and specificity of 82% were found. When CRP exceeds 120 mg/L on postoperative day 5, additional investigations should be considered.
